# Cell-autonomous role of GFRα1 in the development of olfactory bulb GABAergic interneurons

**DOI:** 10.1242/bio.033753

**Published:** 2018-05-01

**Authors:** Sabrina Zechel, Diana Fernandez-Suarez, Carlos F. Ibáñez

**Affiliations:** 1 Department of Cell and Molecular Biology, Karolinska Institute, Stockholm 17177, Sweden; 2 Department of Physiology, National University of Singapore, Singapore 117597, Singapore; 3 Life Sciences Institute, National University of Singapore, Singapore 117456, Singapore; 4 Stellenbosch Institute for Advanced Study, Wallenberg Research Centre at Stellenbosch University, Stellenbosch 7600, South Africa

**Keywords:** Cell migration, RMS, SVZ

## Abstract

GFRα1, a receptor for glial cell line-derived neurotrophic factor (GDNF), is critical for the development of the main olfactory system. The olfactory bulb (OB) of Gfra1 knockout mice shows significant reductions in the number of olfactory sensory neurons, mitral and tufted cells, as well as all major classes of OB GABAergic interneurons. However, the latter do not express significant levels of GFRα1, leaving the mechanism of action of GFRα1 in OB interneuron development unexplained. Here we report that GFRα1 is highly expressed in the precursor cells that give rise to all major classes of OB interneurons, but is downregulated as these neurons mature. Conditional ablation of GFRα1 in embryonic GABAergic cells recapitulated the cell losses observed in global Gfra1 knockouts at birth. GFRα1 was also required for the sustained generation and allocation of OB interneurons in adulthood. Conditional loss of GFRα1 altered the migratory behaviour of neuroblasts along the rostral migratory stream (RMS) as well as RMS glial tunnel formation. Together, these data indicate that GFRα1 functions cell-autonomously in subpopulations of OB interneuron precursors to regulate their generation and allocation in the mammalian OB.

## Abstract (precis)

**Summary:** Our data indicate that GFRα1 functions cell-autonomously in subpopulations of OB interneuron precursors to regulate their generation and allocation in the mammalian OB.

## INTRODUCTION

In the mammalian forebrain, most subpopulations of GABAergic interneurons originate in distant neurogenic areas and migrate to their final locations following trajectories that are, for the most part, tangential to the brain surface. In the developing olfactory system, OB interneuron precursors are generated in the lateral ganglionic eminences, subventricular zone (SVZ) and septum of the embryonic forebrain, and migrate rostrally to the OB through a cell migration pathway known as the rostral migratory stream (RMS) (Lois and Alvarez-Buylla, 1994; Luskin, 1993, 1998; Wichterle et al., 2001). In the glomerular layer (GL) of the OB, interneurons expressing tyrosine hydroxylase (TH), calbindin (CB) and calretinin (CR) are generated sequentially in waves during early embryonic stages, later embryonic stages and postnatal stages, respectively (Batista-Brito et al., 2008). In the mouse embryo, the LGE and the septum were reported to give rise to different types of OB interneurons. While the septum generated mostly GL CR-positive cells, the LGE produced all major GL subpopulations, as well as CR interneurons destined to the granule cell layer (GR) (Qin et al., 2017). At birth, the majority of cells in the GR are CR-positive (Batista-Brito et al., 2008). OB GABAergic interneurons are one of the few neuronal subpopulations that is continuously renewed throughout life from progenitors located in the adult SVZ (Altman et al., 1969; Lledo et al., 2006). Within the adult SVZ, there is considerable heterogeneity and the location of progenitor cells has been shown to determine the types of OB interneurons they generate (Azim et al., 2012; Merkle et al., 2007). In the mouse, it has more recently been shown that the majority of progenitor cells in the adult SVZ (i.e. B cells) are produced during midembryogenesis, and remain quiescent until they become reactivated postnatally (Fuentealba et al., 2015). Despite significant progress, the molecular signals that control the generation, migration, allocation and differentiation of GABAergic interneurons destined for the OB are incompletely understood.

The neurotrophic factor GDNF was initially discovered as a survival factor for midbrain dopaminergic neurons (Lin et al., 1993). It signals by binding to the GPI-anchored receptor GFRα1 (GDNF family receptor alpha 1) in complex with either the receptor tyrosine kinase RET (Treanor et al., 1996; Trupp et al., 1996) or the neural cell adhesion molecule (NCAM) (Paratcha et al., 2003). GFRα1 is therefore an essential component of functional GDNF receptors. In the olfactory system, our laboratory has reported GFRα1 expression in immature olfactory sensory neurons (OSNs) and olfactory ensheathing cells (OECs) of the olfactory epithelium (OE) (Marks et al., 2012). In the OB, GFRα1 was found in projection neurons, namely mitral and external tufted cells, but was largely excluded from GABAergic interneurons (Marks et al., 2012). Knockout mice lacking GFRα1 showed reduced numbers of OSNs, projection neurons, and all major OB interneuron subpopulations, including those marked by expression of TH, CB and CR (Marks et al., 2012). Thus, GFRα1 would appear to play a role in OB interneuron development or maintenance even though it is not itself expressed at mature stages in those cells. Loss of GFRα1 could affect OB interneuron development non-cell-autonomously, as an indirect consequence of defects in OSNs and projection neurons. Alternatively, GFRα1 may initially be required for the development of OB interneuron precursors, but later downregulated in mature OB interneurons. GFRα1 colocalizes with NCAM in RMS cells (Paratcha et al., 2003) and GDNF displays chemoattractant activities towards these cells in cell culture experiments (Paratcha et al., 2006). However, the *in vivo* physiological relevance of those observations has been unclear.

Here, using conditional deletion of GFRα1, we show that this receptor functions transiently and cell-autonomously in subpopulations of OB interneuron precursors to regulate their migration to the OB. We provide evidence showing that selective loss of GFRα1 in GABAergic precursors affects RMS glial tube formation and induces premature neuroblast differentiation, leading to losses in all major subpopulations of OB interneurons.

## RESULTS

### GFRα1 expression in OB GABAergic interneuron precursors of the embryonic septum, olfactory primordium and adult SVZ

**Fig. 1.**
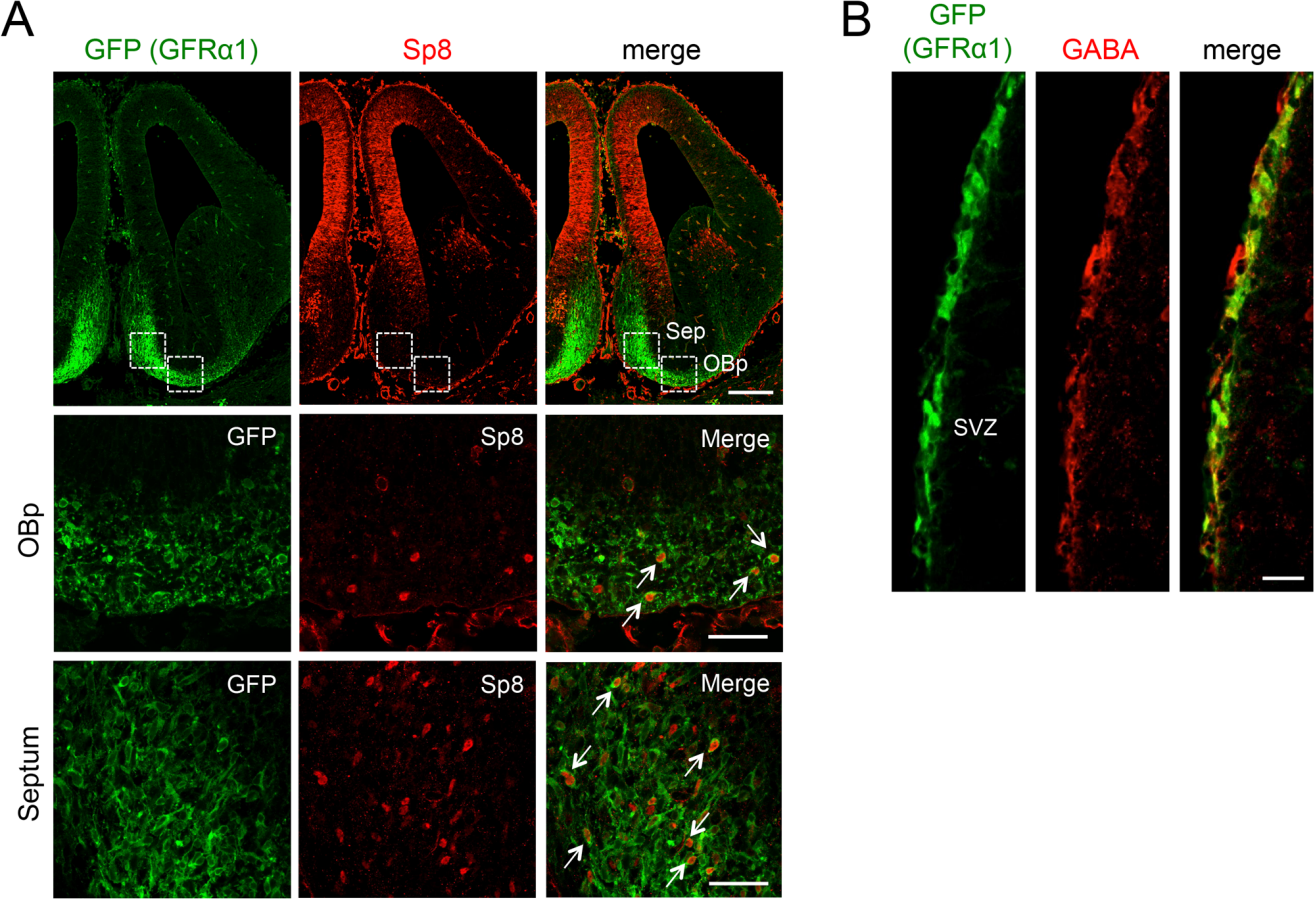
**GFRα1 expression in OB GABAergic interneuron precursors of the embryonic septum and adult subventricular zone (SVZ).** (A) Expression of GFRα1 (green, visualised as GFP expression driven from the R1CG locus after EIIa^Cre^-mediated recombination) and Sp8 (red) detected by immunohistochemistry in cells of the olfactory primordium (OBp) and septum (sep) of E12.5 mouse embryos. The two lower rows display higher magnification images of the areas in septum and OBp indicated in the upper row. In four biological replicates, 65% of Sp8^+^ cells were also GFP^+^ in septum, and 35% in the OBp (arrows). OBp, olfactory primordium; Sep, septum. Scale bars: 200 µm (upper row), 40 µm (two lower rows). (B) Expression of GFRα1 (green, visualised as GFP) and GABA (red) detected by immunohistochemistry in the SVZ of the lateral ventricle in 7-week-old *R1CG*^fx/+^;*EIIa*^Cre^ mice. In five biological replicates, 80% of GABA^+^ cells were also GFP^+^. Scale bar: 30 µm.

The precursors of OB GABAergic interneurons are generated in the lateral ganglionic eminence (LGE), septum and olfactory primordium (OBp) during early embryonic stages and in the subventricular zone (SVZ) at later embryonic stages and throughout adulthood (Lois and Alvarez-Buylla, 1994; Luskin, 1993, 1998). In the embryonic septum and LGE, precursor cells expressing the Sp8 transcription factor can give rise to OB CR-expressing cells (Waclaw et al., 2006; Young et al., 2007). Previous studies had indicated that GFRα1 is not expressed in the LGE (Canty et al., 2009; Pozas and Ibáñez, 2005). We used *R1CG*^fx/fx^ mice, which have been engineered to express green fluorescent protein (GFP) from the *Gfra1* locus upon Cre-mediated recombination (Uesaka et al., 2007). *R1CG*^fx/+^;*EIIa*^Cre^ mice express GFP in all GFRα1-postitive cells and retain one functional *Gfra1* allele. At embryonic day 12.5 (E12.5), GFP was detected in cells of the OBp and developing septum, several of which also expressed Sp8 (Fig. 1A). These results confirm that GFRα1 is expressed in subpopulations of Sp8^+^ precursors localised to the septum and OBp. In order to identify cell precursors of OB interneurons in postnatal adult SVZ, we performed immunohistochemistry on sections through the lateral wall of the lateral ventricle and detected significant overlap between GFP and GABA (Fig. 1B). Together, these results indicated that GFRα1 is expressed in subpopulations of precursors of OB GABAergic interneurons at both embryonic and adult stages.

### Precursors and neuroblasts expressing GFRα1 contribute to all major subpopulations of GABAergic interneurons in the newborn and adult OB

**Fig. 2.**
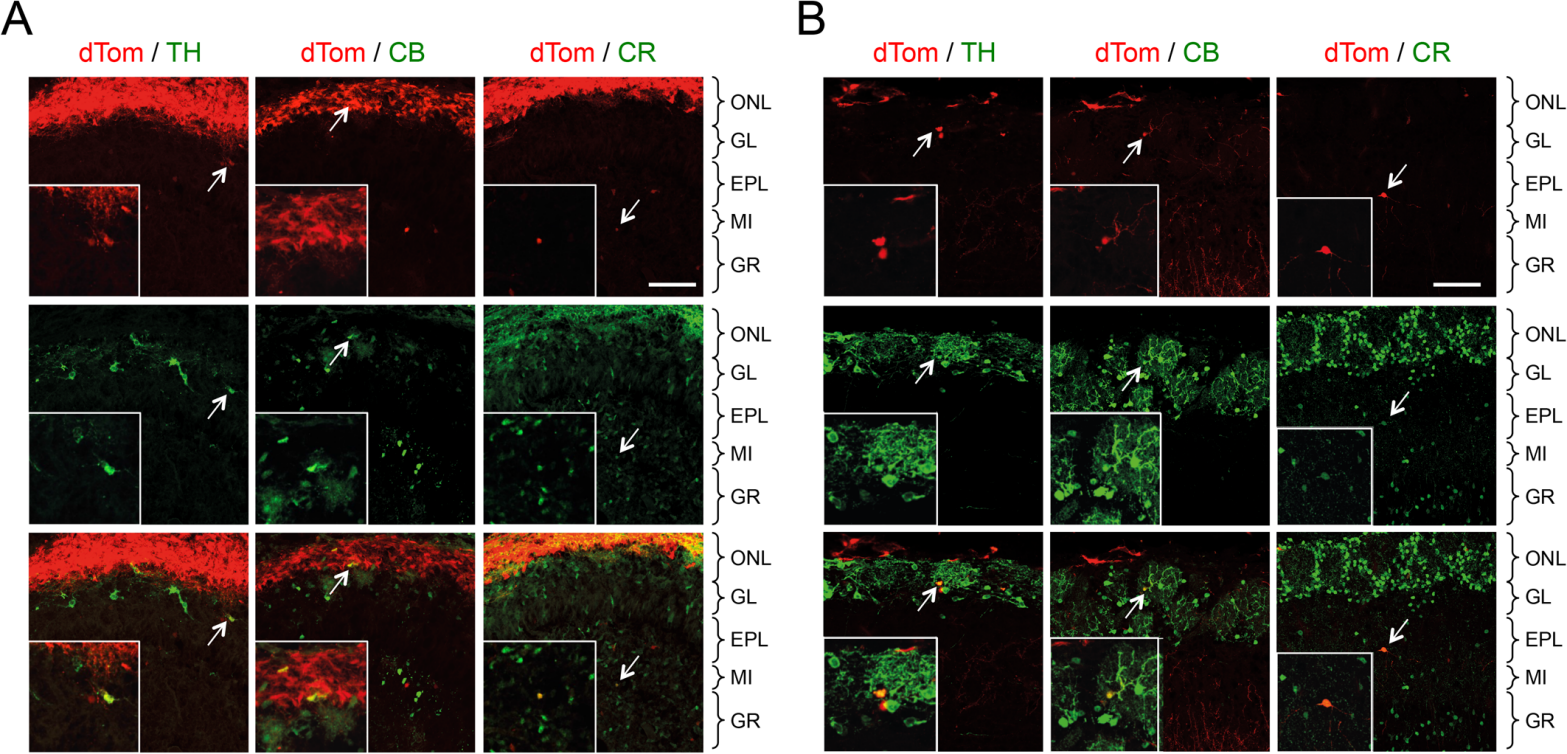
**Precursors cells** **expressing GFRα1 contribute to all major subpopulations of GABAergic interneurons in the newborn and adult OB.** (A) Expression of dTomato (dTom) in the OB of newborn (P0) *Gfra1*^CreERT2/+^;dTom mice injected with Tamoxifen at E10.5 and E11.5. The lower panels show overlap of dTom labelled cells with OB GABAergic interneuron markers tyrosine hydroxylase (TH), Calbindin and Calretinin (arrows). Inserts show higher magnification of double-positive cells. The different OB layers are indicated on the right. In six biological replicates, 25% of TH^+^, 11% of CB^+^ and 15% of CR^+^ cells were also dTom^+^. ONL, olfactory nerve layer; GL, glomerular layer; EPL, external plexiform layer; MI, mitral cell layer; GR, granule cell layer. Scale bar: 50 µm. (B) Expression of dTomato (dTom) in the OB of P56 *Gfra1*^CreERT2/+^;dTom mice injected with Tamoxifen at P21, P22 and P23. The lower panels show overlap of dTom labelled cells with OB GABAergic interneuron markers tyrosine hydroxylase (TH), Calbindin and Calretinin (arrows). Inserts show higher magnification of double-positive cells. The different OB layers are indicated on the right. In six biological replicates, 5% of TH^+^, 3% of CB^+^ and 30% of CR^+^ cells were also dTom^+^. ONL, olfactory nerve layer; GL, glomerular layer; EPL, external plexiform layer; MI, mitral cell layer; GR, granule cell layer. Scale bar: 50 µm.

Next, we assessed the contribution of precursor cells expressing GFRα1 to the major classes of OB interneurons during both embryonic development and in the adult. We performed inducible genetic fate mapping by treating *Gfra1*^CreERT2/+^;dTom pregnant females with Tmx at E10.5 and E11.5 and analysed the phenotypes of OB dTom-positive cells at birth (P0). We observed dTom-positive cells in the granule cell and glomerular layers of the newborn OB as well as a prominent dTom signal in the olfactory nerve layer (ONL) (Fig. 2A), in agreement with our previous studies (Marks et al., 2012). In the GL, dTom-positive cells were seen to co-express TH and CB, while in the GR, we detected co-expression with CR (Fig. 2A). Because of the inherent limitations of Tmx-directed recombination, the contribution of precursor cells expressing GFRα1 to these OB interneuron types could not be quantitatively determined from this analysis. Nevertheless, these results indicate that GFRα1-expressing precursors in the embryo can give rise to all major classes of OB interneurons at birth. In order to study the progeny of adult GFRα1-expressing precursor cells, we injected Tmx at P21, P22 and P23 and examined their OB fates at P56. Similar to embryonic precursors, we found that adult neuroblasts expressing GFRα1 can give rise to interneurons expressing TH, CB or CR in the adult OB (Fig. 2B). Given that most OB interneurons do not themselves express GFRα1 (Marks et al., 2012), we conclude that this receptor is transiently expressed in subpopulations of precursor cells and neuroblasts that later gives rise to OB interneurons through embryonic development and in the adult. GFRα1 may contribute to different aspects of the development of OB interneuron precursors, such as proliferation, differentiation or migration, before being downregulated in mature OB interneurons.

### Cell-autonomous loss of GABAergic interneurons in the OB of newborn and adult conditional GFRα1 mutants

**Fig. 3.**
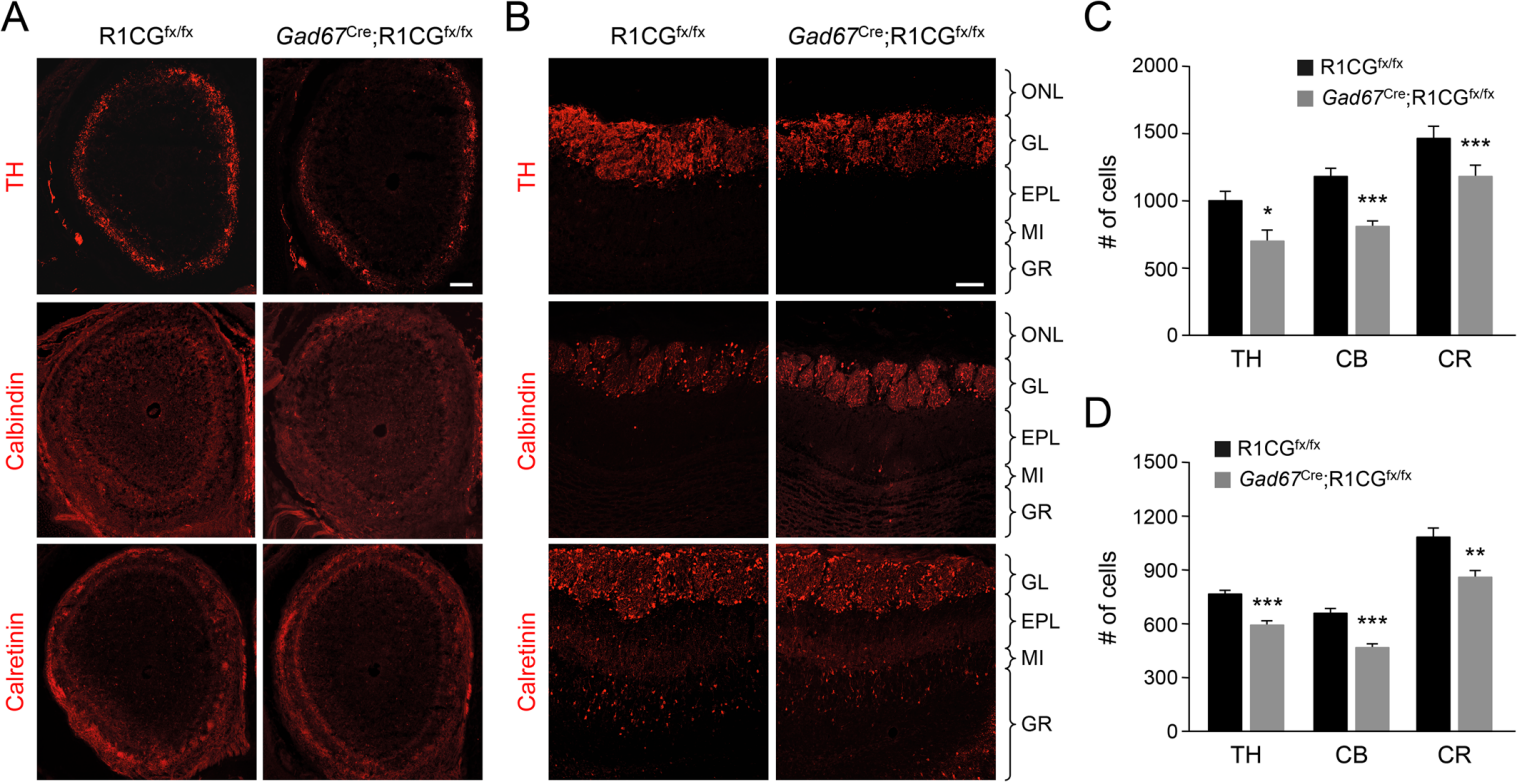
**Cell-autonomous loss of GABAergic interneurons in the OB of newborn and adult conditional GFRα1 mutants.** (A) Representative images of the OB of newborn *Gad67*^Cre^;R1CG^fx/fx^ conditional mutant and R1CG^fx/fx^ control mice immunostained for markers of OB GABAergic interneurons: tyrosine hydroxylase (TH), Calbindin and Calretinin. Scale bar: 100 µm. (B) Representative images of the OB of P56 *Gad67*^Cre^;R1CG^fx/fx^ conditional mutant and R1CG^fx/fx^ control mice along the medial surface immunostained for markers of OB GABAergic interneurons: tyrosine hydroxylase (TH), Calbindin and Calretinin. Scale bar: 100 µm. (C) Quantification of the number of cells expressing TH, Calbindin (CB) and Calretinin (CR) in the OB of newborn *Gad67*^Cre^;R1CG^fx/fx^ conditional mutants and R1CG^fx/fx^ control mice. The values represent total number of cells in fields encompassing the entire OB. *N*=6 mice per group; *P<0.05; ***P<0.0005. (D) Quantification of the number of cells expressing TH, Calbindin (CB) and Calretinin (CR) in the OB of P56 *Gad67*^Cre^;R1CG^fx/fx^ conditional mutants and R1CG^fx/fx^ control mice. The values represent number of cells counted in the glomerular layer of the medial surface of the OB (see the Materials and Methods for details). *N*=6 mice per group; **P<0.005; ***P<0.0005. ONL, olfactory nerve layer; GL, glomerular layer; EPL, external plexiform layer; MI, mitral cell layer; GR, granule cell layer.

Our finding of GFRα1 expression in embryonic and adult precursors of OB GABAergic neurons opened the possibility that a cell-autonomous, albeit transient, function of GFRα1 in these precursors may regulate the final number of OB GABAergic interneurons. As the precursors of all TH- and CB-positive periglomerular cells, as well as the majority of CR-positive granule neurons, are also positive for GAD67 (Kosaka and Kosaka, 2007; Sawada et al., 2011), we crossed mice expressing Cre recombinase from the *Gad67* locus (Tolu et al., 2010) with *R1CG*^fx/fx^ mice, and examined the effects of GFRα1 ablation in GABAergic cell precursors on the complement of all the major types of OB interneurons. Efficient recombination Cre-mediated was verified by immunostaining of GFRα1 in sections of the septal area of 7-week-old *Gad67*^CRE^;*R1CG*^fx/fx^ and *R1CG*^fx/fx^ mice (Fig. S1). At birth, *Gad67*^CRE^;*R1CG*^fx/fx^ mice showed 20–30% reduction in TH, CB and CR positive cells (Fig. 3A,C). This difference was maintained in the 8-week-old OB of the mutants (Fig. 3B,D), indicating that loss of OB GABAergic interneurons cannot be compensated by newly generated interneurons during adulthood. Together, these data suggested that GFRα1 regulates the development of OB GABAergic interneurons cell-autonomously in GABAergic precursors.

In order to more directly assess the possibility of non-cell-autonomous effects of GFRα1 on the complement of OB GABAergic interneurons, we investigated the impact of GFRα1 loss in OSNs and projection neurons. Primary olfactory axons have been implicated in regulating early neurogenesis in the OB (de Carlos et al., 1995; Gong and Shipley, 1995), and there is also evidence that OE activity can influence the proliferation of precursor cells in the SVZ (Ma et al., 2009; Mandairon et al., 2003). Likewise, glutamate input from excitatory neurons has been shown to affect OB interneuron function (Abraham et al., 2010). At 8 weeks of age, mice lacking GFRα1 in OSNs (*γ8TTA-TetO*^Cre^;*R1CG*^fx/fx^) displayed a reduction in mature, OMP-positive OSNs that was comparable to the losses that we previously reported in the global *Gfra1* knockout (Marks et al., 2012) (Fig. S2A,B). However, no reduction in GABAergic interneurons could be detected in either the newborn or adult OB of these mice (Fig. S3A,B). Similarly, mice lacking GFRα1 in OB excitatory neurons (*Pcdh21*^Cre^;*R1CG*^fx/fx^) also showed a normal complement of OB GABAergic interneurons at both ages (Fig. S3C,D). We note that no significant reduction in the numbers of either mitral or tufted cells could be detected in the OB of these mice (Fig. S4A–C), suggesting that the loss of OB excitatory neurons previously observed in the global knockout may be non-cell-autonomous and secondary to other defects in the olfactory system of those mice. In summary, together with our genetic fate mapping studies, these results support a cell-autonomous role for GFRα1 in the regulation of OB GABAergic interneuron development.

### Continued requirement of GFRα1 for the maintenance of the normal complement of OB interneurons in adult mice

**Fig. 4.**
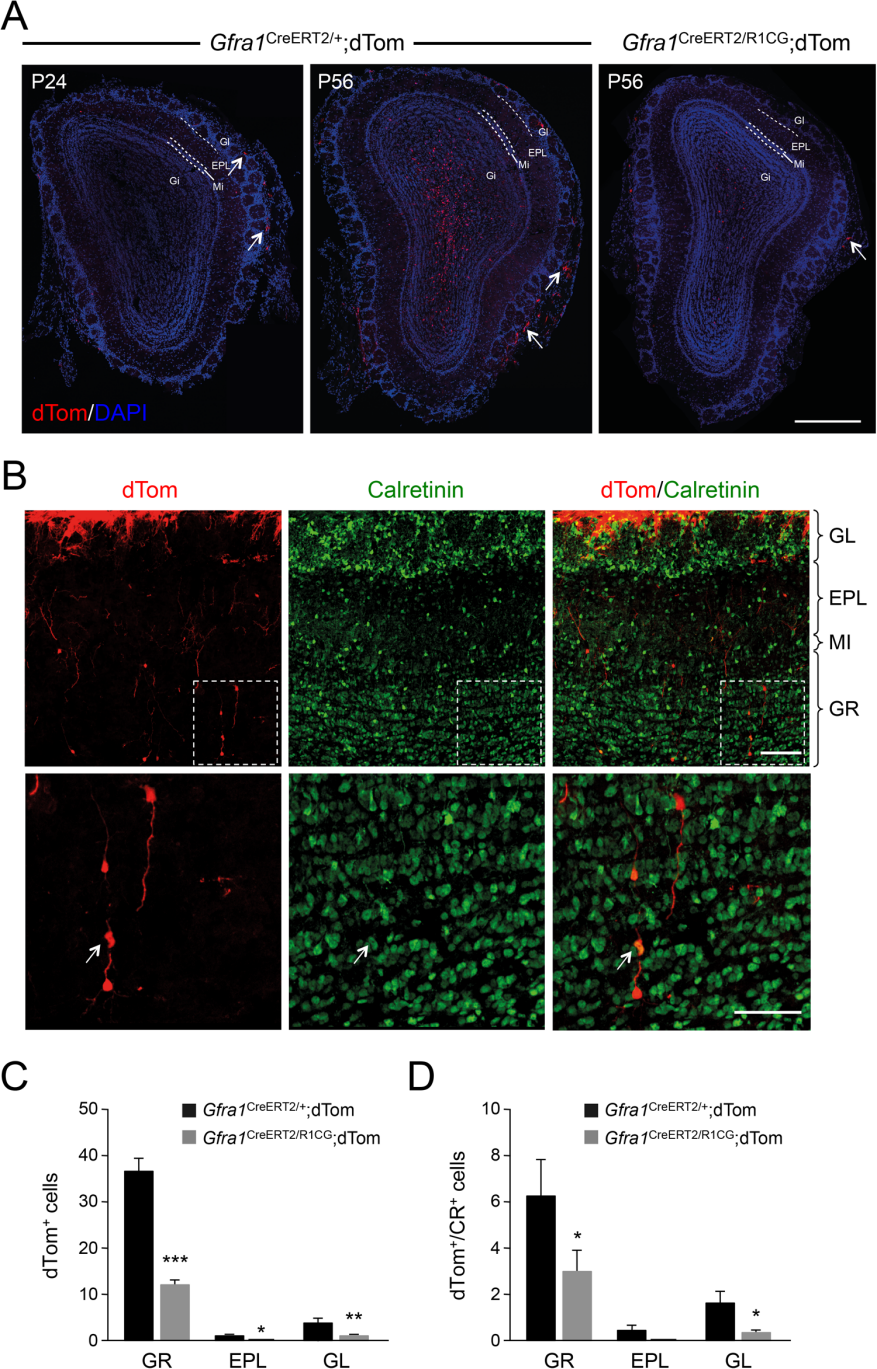
**Continued requirement of GFRα1 for the maintenance of the normal complement of OB interneurons in adult mice.** (A) Representative coronal images of the OB of *Gfra1*^CreERT2/+^;dTom and *Gfra1*^CreERT2/R1CG^;dTom mice of the ages indicated after Tmx induced recombination at P21, P22 and P23. Arrows indicate ensheathing cells of the olfactory nerve layer. Scale bar: 500 µm. (B) Expression of dTom and Calretinin in the OB of P56 *Gfra1*^CreERT2/+^;dTom mice after Tmx induced recombination at P21, P22 and P23. Inserts in the second row show higher magnification of boxed areas. In four biological replicates, 20% of CR^+^ cells were also dTom^+^ (arrow). Scale bars: 50 µm (insets 25 µm). (C,D) Quantification of total (C) dTom-positive and (D) dTom/Calretinin double-positive cells in the OB of P56 *Gfra1*^CreERT2/+^;dTom and *Gfra1*^CreERT2/R1CG^;dTom mice after Tmx induced recombination at P21, P22 and P23. *N*=4 mice per group; *P<0.05; **P<0.005; ***P<0.0005. GL, glomerular layer; EPL, external plexiform layer; MI, mitral cell layer; IPL, internal plexiform layer; GR, granule cell layer.

Next, we investigated whether GFRα1 is also required during postnatal stages for the maintenance of the normal complement of OB interneurons in the adult. To this end, we used the *Gfra1*^CreERT2^ allele and a Cre-dependent dTomato reporter to follow the fate of GABAergic interneurons generated in a mature olfactory system (between P21 and P56). We injected Tmx in 3-week-old *Gfra1*^CreERT2/+^;dTom mice (which are heterozygous for the wild-type *Gfra1* allele) during three consecutive days and assessed dTom-positive cells in the OB at P24 and at P56. At P24, one day after the last Tmx injection, a few labelled cells could be observed in the olfactory nerve layer, likely corresponding to ensheathing cells [see Marks et al. (2012)], while no significant labelling could be detected in the GR or GL (Fig. 4A, left panel). At P56, on the other hand, numerous dTom-positive cells could be observed in the GL, and several labelled cells could also be seen in the glomerular layer and underlying external plexiform layer (Fig. 4A, centre panel). This is in agreement with observations indicating that SVZ neuroblasts take 3–4 weeks to reach the GL (Lemasson et al., 2005). Importantly, a significant loss of dTom-positive cells was seen across all layers in the OB of compound mutant *Gfra1*^CreERT2/R1CG^;dTom mice, which lost GFRα1 expression from 3 weeks of age onwards after Tmx injection and Cre-mediated recombination (Fig. 4A, right panel). A quantitative analysis revealed prominent losses among CR-positive cells in the granule cell layer of these mice (Fig. 4B–D), which is the preferred fate adopted by postnatal neuroblasts (Lemasson et al., 2005; Batista-Brito et al., 2008).

### Loss of GFRα1 in OB GABAergic cell precursors affects neuroblast migration, differentiation and glial tunnel formation in the RMS

The loss of GABAergic interneurons in the OB of *Gad67*^CRE^;*R1CG*^fx/fx^ mice could be due to defects in proliferation or survival of GABAergic precursor cells, or in the migration or differentiation of RMS neuroblasts, all of which express GFRα1. We assessed cell proliferation in the embryonic septum at the peak of OB interneuron generation (E16.5) and in the adult (P56) SVZ by BrdU injections. We did not detect any difference in the extent of BrdU incorporation between the mutants and the controls (Fig. S5). We also quantified apoptotic cell death by staining for cleaved Caspase-3 combined with presence of pycnotic nuclei (assessed by DAPI staining) in conditional GFRα1 mutants and control mice. We could not detect a significant increase in cell death at neurogenic sites, nor along the migratory pathway, nor in the OB in either newborn or adult (P56) conditional GFRα1 mutant mice compared to controls (Fig. S6). Together, these results indicated that loss of GFRα1 in GABAergic precursors does not affect their proliferation or survival.

**Fig. 5.**
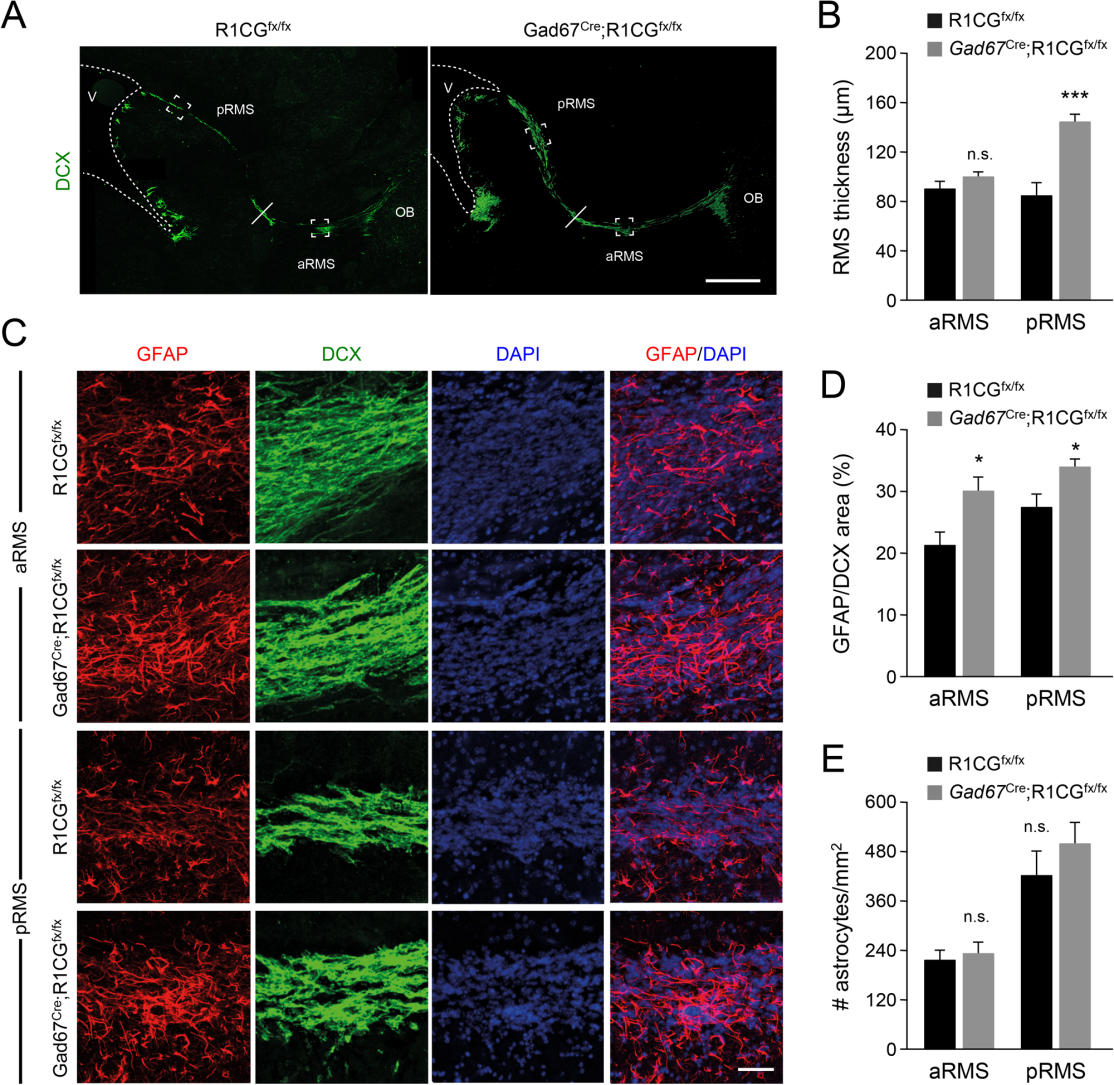
**RMS abnormalities in conditional mutant mice lacking GFRα1 in OB GABAergic cell precursors.** (A) Representative images of DCX expression in a sagittal section of the RMS of P56 *Gad67*^Cre^;R1CG^fx/fx^ conditional mutant and R1CG^fx/fx^ control mice. The unbroken line marks the border between aRMS and pRMS. The ventricle (v) and OB are indicated. aRMS, anterior RMS; pRMS, posterior RMS. Scale bar: 500 µm. (B) Quantification of aRMS and pRMS thickness at the positions boxed in panel A. *N*=8 mice per group. n.s., not significantly different; ***P<0.0005. (C) GFAP (red), DCX (green) and DAPI (blue) staining of sagittal sections through the anterior (aRMS) and posterior (pRMS) RMS of P56 *Gad67*^Cre^;R1CG^fx/fx^ conditional mutant and R1CG^fx/fx^ control mice. Scale bar: 25 µm. (D) Percentage of DCX area covered by GFAP-positive astroglial structures in the aRMS and pRMS of P56 *Gad67*^Cre^;R1CG^fx/fx^ conditional mutant and R1CG^fx/fx^ control mice. *N*=8 mice per group; *P<0.05. (E) Quantification of astrocyte number per unit of DCX^+^ area (assessed by DAPI nuclei within GFAP^+^ structures) in the aRMS and pRMS of P56 *Gad67*^Cre^;R1CG^fx/fx^ conditional mutant and R1CG^fx/fx^ control mice. *N*=8 mice per group. n.s., not significantly different.

**Fig. 6.**
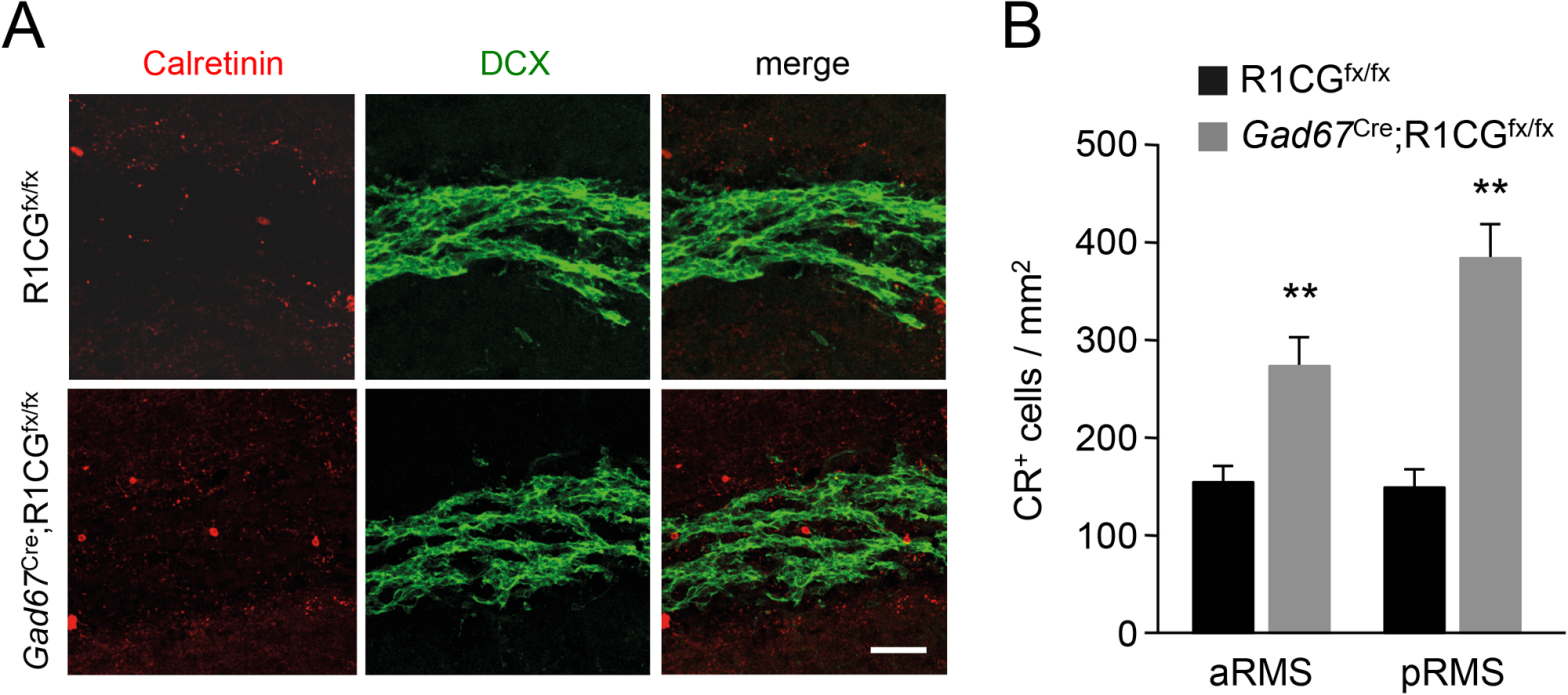
**Premature differentiation of RMS neuroblasts in conditional mutant mice lacking GFRα1 in OB GABAergic cell precursors.** (A) Immunostaining for Calretinin (red) and DCX (green) in the posterior RMS of P56 *Gad67*^Cre^;R1CG^fx/fx^ conditional mutant and R1CG^fx/fx^ control mice. Scale bar: 50 µm. (B) Quantification of Calretinin positive cells per unit DCX^+^ area in the RMS of P56 *Gad67*^Cre^;R1CG^fx/fx^ conditional mutant and R1CG^fx/fx^ control mice. *N*=6 mice per group. **P<0.005.

Next, we turn our attention to the RMS of *Gad67*^CRE^;*R1CG*^fx/fx^ mice. At 8 weeks of age (P56), DCX staining of the RMS revealed a broader posterior RMS (pRMS) in the mutants (Fig. 5A,B). A similar phenotype has been reported in global knockouts of *Ncam* (Chazal et al., 2000); and was attributed to abnormal neuroblast migration in the RMS. As they leave the SVZ and enter the posterior RMS, neuroblasts accumulate in this region. In the *Ncam* mutants, the RMS enlargement is accompanied by an increase in GFAP-positive astroglial structures along the RMS, without a change in astrocyte proliferation or number (Chazal et al., 2000). Astrocytes ensheathing the RMS are thought to provide guidance to migrating RMS neuroblasts (Alvarez-Buylla and Lim, 2004). We assessed astroglial coverage in the RMS of *Gad67*^CRE^;*R1CG*^fx/fx^ mice by quantifying the area covered by GFAP immunostaining within the region stained by DCX in anterior and posterior RMS regions. We detected a significant increase in the mutants compared to controls (Fig. 5C,D) without a change in astrocyte number (Fig. 6E), suggesting enlarge astrocyte area in the RMS of the mutants. In addition, the RMS of *Gad67*^CRE^;*R1CG*^fx/fx^ mice showed an increased number of CR expressing cells relative to DCX-positive area (Fig. 6A,B), suggesting premature differentiation of RMS neuroblasts in the mutant, a phenotype that has also been observed in *Ncam* deficient mice (Röckle and Hildebrandt, 2016).

**Fig. 7.**
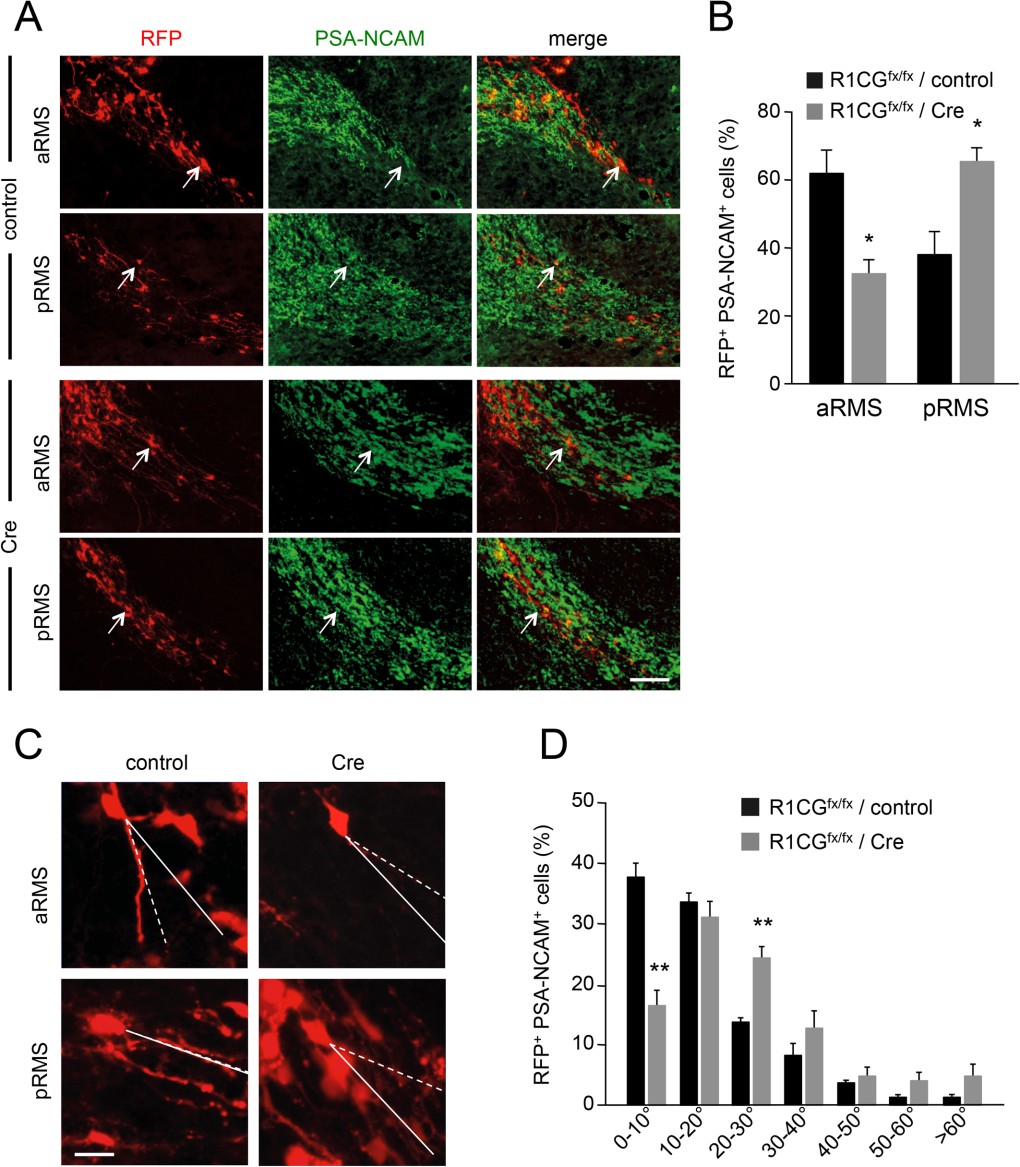
**Abnormal neuroblast migration in the RMS of conditional mutant mice lacking GFRα1 in OB GABAergic cell precursors.** (A) Representative images of RFP (red, marking all electroporated cells) and PSA-NCAM (green, marking RMS neuroblasts) expression in the anterior and posterior RMS of P21 R1CG^fx/fx^ mice after ventricular electroporation of Cre-expressing or control plasmids at P0. Arrows indicate double-positive cells. Scale bar: 50 µm. (B) Quantification of the proportion of RFP/PSA-NCAM double-positive cells in the anterior and posterior RMS of P21 R1CG^fx/fx^ mice after ventricular electroporation of Cre-expressing or control plasmids normalised to the total number of double-positive cells in the RMS. *N*=7 mice per group. *P<0.05. (C) Representative images of RFP^+^ cells in the anterior and posterior RMS of P21 R1CG^fx/fx^ mice after ventricular electroporation of Cre-expressing or control plasmids at P0. The angles between the leading process of individual, electroporated neuroblasts (dashed lines) and the main direction of the RMS (solid lines) are indicated. Scale bar: 10 µm. (D) Analysis of the angle of the leading process with respect to the lateral walls of the RMS (as defined by PSA-NCMA staining) in RFP/PSA-NCAM double-positive cells of the RMS of P21 R1CG^fx/fx^ mice after ventricular electroporation of Cre-expressing or control plasmids. The analysis included both anterior and posterior RMS. *N*=4 (control); *N*=7 (Cre) mice per group. *P*=**, <0.005.

In order to investigate neuroblast migration in the RMS, we used *in vivo* electroporation to introduce a fluorescent reporter (RFP) together with Cre recombinase in the ventricular zone of newborn *R1CG*^fx/fx^ mice. This method resulted in efficient electroporation of GFAP-positive type B stem cells and Ascl1/MASH1-positive type C transit amplifying cells in the SVZ (Fig. S7A,B). At 3 weeks of age (P21), *R1CG*^fx/fx^ neuroblasts that had undergone Cre-mediated recombination (i.e. double-positive for RFP and PSA-NCAM) accumulated in the posterior RMS to a larger extent than in controls that did not receive Cre (Fig. 7A,B), indicating a slower migration along the RMS after loss of GFRα1 expression. We also measured the angle between the leading process of individual, electroporated neuroblasts and the main direction of the RMS (see the Materials and Methods section). We found that Cre-mediated loss of GFRα1 significantly increased the angle of the leading process in migrating neuroblasts in the RMS (Fig. 8C,D), in agreement with abnormal migratory behaviour. Together, these results indicate altered neuroblast migration, premature differentiation and abnormal glial tunnel formation in the RMS of mice lacking GFRα1 in OB GABAergic cell precursors.

## DISCUSSION

In the present study, we have shown that GFRα1 is expressed in GABAergic precursors contributing to all classes of OB interneurons and that conditional loss of GFRα1 in GABAergic cells, during development or in adulthood, results in reduced numbers of all major OB interneurons subtypes. In addition, the RMS of conditional mutants shows abnormal neuroblast migration, differentiation and increased astroglial reactivity. These results indicate a cell-autonomous requirement of GFRα1 in the cell lineage that gives rise to OB interneurons.

OB interneurons are thought to be mainly generated in the LGE and septum from E14.5 onwards during embryonic development (Pencea and Luskin, 2003; Wichterle et al., 2001). It has also been reported that some OB interneurons may be generated in the olfactory primordium from E11 (Hinds, 1968; Vergaño-Vera et al., 2006). We have detected expression of GFRα1 in subpopulations of cells in the septum, olfactory primordium and developing OB, but not in the LGE, of E12.5 embryos. Some of those cells also expressed Sp8, a known marker of the precursors of OB CR-positive cells. These observations suggest a significant level of heterogeneity among precursors of OB GABAergic interneurons from early stages of embryonic development. In the adult, interneurons destined for the OB originate from the SVZ, and the majority of those cells differentiate into granule cells (Lemasson et al., 2005; Batista-Brito et al., 2008). This is in agreement with our observation that the majority of OB interneurons deriving from GFRα1-expressing SVZ precursors become CR-positive cells of the granule cell layer. Depending on their location, granule cells can differ in their connectivity and functional properties (Merkle et al., 2014). The fact that granule cells derived from GFRα1^+^ precursors appeared distributed across superficial, intermediate and deep layers of the GL suggests that they are themselves functionally heterogeneous. The expression of GFRα1 in migratory precursors, but not in mature OB GABAergic interneurons, indicates that this receptor is downregulated during the final maturation of these cells. Transient expression of GFRα1 by migratory precursors of GABAergic neurons seems to be a common property of this type of cell as it has also been observed in GABAergic precursors of the MGE that give rise to cortical interneurons (Canty et al., 2009; Pozas and Ibáñez, 2005), immature molecular layer interneurons of the cerebellum (Sergaki et al., 2017) and in migratory precursors of Purkinje cells (Sergaki and Ibáñez, 2017). It remains unclear why or how GABAergic precursor cells downregulate expression of GFRα1 as they develop, but it may be related to their cessation of cell migration and incorporation into mature neuronal circuits.

The extent of the loss of GABAergic interneurons in the OB of newborn and adult *Gad67*^CRE^;*R1CG*^fx/fx^ mutant mice (approximately 20–30%) was comparable to that previously observed in global *Gfra1* knockout mice (Marks et al., 2012). Interestingly, the loss of mature OSNs observed in *γ8TTA-TetO*^Cre^;*R1CG*^fx/fx^ mice was not accompanied by a reduction in OB GABAergic interneurons in these mutants. Thus, although OE activity has been shown to influence early OB neurogenesis (de Carlos et al., 1995; Gong and Shipley, 1995), there does not seem to be a simple relationship between the complements of OSN and OB interneurons. Finally, removal of GFRα1 in mitral and tufted cells did not affect the number of GABAergic neurons in the OB. Together, these results support a cell-autonomous function for GFRα1 in the GABAergic cell lineage that gives rise to OB interneurons.

Together with RET, GFRα1 has been shown to contribute to GDNF-mediated cell survival. Among GABAergic cells, for example, we have recently reported that cerebellar molecular layer interneurons depend upon expression of GFRα1 and RET during development for their survival in response to GDNF derived from Purkinje cells (Sergaki et al., 2017). In cells that do not express RET, GFRα1 has been implicated in cell migration, either in partnership with NCAM, as in the case of Purkinje cell precursors (Sergaki and Ibáñez, 2017), or together with other yet unknown receptors, as in the GABAergic precursors derived from the MGE (Perrinjaquet et al., 2011; Pozas and Ibáñez, 2005). In this study, we have shown that the specific loss of GFRα1 in GABAergic cells does not alter their proliferation or survival in neurogenic areas known to give rise to OB GABAergic interneurons, namely the embryonic septum and adult SVZ. On the other hand, we have presented several lines of evidence that indicate a role for GFRα1 in the migration and early differentiation of these cells. First, the posterior RMS of conditional mutants lacking GFRα1 in GABAergic cells is enlarged, a phenotype that has also been described in mice lacking NCAM (Chazal et al., 2000) and which is thought to result from aberrant migration of these cells in the initial segments of the RMS. Second, GFRα1-expressing SVZ precursors labelled by Cre-mediated recombination at their site of origin also accumulate in the posterior RMS at the expense of more distal RMS regions, in agreement with a sluggish migratory behaviour. Finally, a proportion of RMS neuroblast in *Gad67*^CRE^;*R1CG*^fx/fx^ mice prematurely differentiated into CR-positive neurons, a phenotype that has also been observed in *Ncam* knockouts (Röckle and Hildebrandt, 2016), and which suggests that GFRα1 may contribute to maintain GABAergic neuroblasts in an immature, migration-prone state. In addition, we observed increased astrocyte area in the RMS of *Gad67*^CRE^;*R1CG*^fx/fx^ mice, suggesting astrocyte activation (a.k.a. astrogliosis by some authors), which has also been reported in *Ncam* mutants (Chazal et al., 2000). As RMS astrocytes do not express GFRα1, this reaction may be an indirect consequence of the abnormal behaviour of RMS neuroblasts in mutant mice lacking GFRα1 in GABAergic cells, and suggest a close interplay between RMS astrocytes and migrating neuroblasts. Although astrocytes are only generated later in development, radial glial cells may be playing a similar scaffold role for early GABAergic cells destined to the OB during embryonic development (Alves et al., 2002).

In summary, our results indicate that transient expression of GFRα1 in OB interneuron precursors plays important roles in a cell-autonomous fashion during development as well as adulthood, thus resolving the unexplained phenotype previously reported in global *Gfra1* mutants. Although the similarities with the phenotypes described in mice lacking NCAM suggest that this cell adhesion molecule serves as GFRα1 co-receptor in the murine RMS, the precise mechanisms by which GFRα1 regulates the migration of neuroblasts to the OB needs further investigation.

## MATERIALS AND METHODS

### Animals

The mouse lines utilised in this study have been described previously and are as follows: (i) conditional *Gfra1* mutants, referred to as *R1CG*^fx/fx^, express GFP from the *Gfra1* locus after Cre-mediated recombination (Uesaka et al., 2007); (ii) *Gad67*^Cre^ (Tolu et al., 2010); (iii) *γ8TTA-tetO*^Cre^ (Nguyen et al., 2007); (iv) *Pcdh21*^Cre^ (Nagai et al., 2005); (v) *EIIa*^Cre^ (Lakso et al., 1996); (vi) *ROSA26*^dTom^ (Madisen et al., 2010); (vii) *Gad*^GFP^ (Tamamaki et al., 2003); and (viii) *Gfra1*^CreERT2^ (Sergaki and Ibáñez, 2017). All mouse lines were bred on a C57BL6 background. Both males and females were used for these studies. The day of the vaginal plug was considered as embryonic day 0.5 (E0.5). Animal protocols were approved by Stockholm’s Norra Djurförsöksetiska Nämnd and are in accordance with the ethical guidelines of the Karolinska Institute.

### Immunohistochemistry and *in situ* hybridization

Embryos and neonatal pups were decapitated and fixed in 4% paraformaldehyde (PFA, Sigma-Aldrich) for 24 h at 4°C. Three- or 8-week-old mice were deeply anaesthetised with isoflurane, and perfused transcardially with ice-cold PBS followed by 4% PFA. All samples were subsequently washed in PBS, cryoprotected in 30% sucrose at 4°C and serially sectioned (12 μm) on a cryostat (Cryostar, NX70, Microm, Bicester, UK). After blocking in 5% serum for 1 h, sections were incubated in the following primary antibodies overnight at room temperature (RT): rabbit anti-CB (D28K, ab1778, Millipore, 1:500), rabbit anti-CR (ab5054, Millipore, 1:500), rabbit anti-TH (ab657012, Millipore, 1:500), chicken anti-GFP (ab13970, Abcam, 1:500), goat anti-GFP (ab6673, Abcam, 1:500), rabbit anti-cleaved Caspase-3 (Asp175, 9661, Cell Signaling Technology, 1:500), guinea pig anti-doublecortin (DCX, ab2253, Millipore, 1:500), goat anti-Sp8 (C18, sc104661,Santa Cruz Biotechnology, 1:500), anti-mouse anti-PSA-NCAM (clone 2-2B, MAB5324, Millipore, 1:500), rabbit anti-GFAP (ab5804, Millipore, 1:500), rabbit anti-ER81 (ab36788-50, Abcam, 1:500), goat anti-OMP (Wako Pure Chemicals, Richmond, USA, 1:500), rabbit anti-GAP43 (Novus Biochemicals, Littleton, USA, 1:500), rabbit anti-Ascl1/MASH1 (ab74065, Abcam, 1:1000), mouse anti-Reelin (MAB5364, Millipore, 1:500), and rat anti-BrdU (347580, AbD Serotec, Hercules, USA, 1:500). After three washes in PBS, slides were incubated with fluorescently labelled secondary antibodies (Jackson ImmunoResearch, 1:500) for 2 h at RT. Slides were then washed in PBS, counterstained with 4′-6-diamidino-2-phenylindole (DAPI) and coverslipped in fluorescent mounting medium (Dako, Glostrup, Denmark). For *in situ* hybridization, a fragment corresponding to the extracellular domain of mouse GFRα1 was amplified by RT-PCR and subcloned by TOPO-TA cloning (Invitrogen). Non-radioactive fluorescent *in situ* hybridization was performed by hybridization overnight at 58°C using a specific antisense riboprobe labelled with biotin-dUTP (Roche Diagnostics, Basel, Switzerland). Sections were processed using tyramide signal amplification (Perkin Elmer, Waltham, USA). Finally, sections were developed with Streptavidin 488 (Millipore, 1:500). Control hybridizations with sense riboprobe did not give any signal (Pozas and Ibáñez, 2005).

### BrdU labelling and Tamoxifen injection

Adult (2-months-old) and E16.5 pregnant animals were injected intraperitoneally with BrdU (100 mg/kg body weight, Sigma-Aldrich) in PBS. After 1 h (adult mice) or 30 min (E16.5) the mice were deeply anaesthetised and perfused transcardially with PBS followed by 4% PFA. Brains were removed, postfixed, cryoprotected and cut as described above. Sections were incubated in 1 M HCI at 45°C for 45 min to denature the DNA before immunohistochemical staining. For tamoxifen administration, time-mated pregnant females were injected intraperitoneally with Tamoxifen (Sigma-Aldrich, T5648) in corn oil at a concentration of 100 mg/kg body weight.

### Electroporation of newborn mouse pups

Electroporation of the ventricle wall in neuronal mouse pups was performed as described (Chesler et al., 2008) after intraventricular injection of plasmids encoding red-fluorescent protein (RFP) with or without a Cre recombinase-encoding plasmid, combined with 0.05% Fast Green (Sigma-Aldrich) as a tracer. Briefly, neonatal pups were shortly anaesthetised by hypothermia and 1–2 µl plasmid solution was injected into each lateral ventricle. Tweezer electrodes were placed horizontally as well as tilted in a 45° angle to each side. A square wave electroporator NEPA21 (Nepagene, Chiba, Japan) was used to deliver 2×10 ms pulses of 175 V for electroporation followed by 3×50 ms pulses of 15 V for transfer (with 50 ms intervals). Three weeks later, the mice were perfused, postfixed and processed for immunohistochemistry as described above.

### Image analysis

Immunofluorescence images were captured with a Carl Zeiss LSM710 confocal microscope (10 µm thick, 20× magnification, z=10). For interneuron counts in the OB, nine representative images were sampled from the anterior, middle and posterior sagittal planes of the OB for each animal within each group. For OB cell counts in newborn mice, confocal images were collected encompassing the entire OB in coronal view. In 8-week-old mice, cells within the glomerular layer of the medial portion of the OB were counted in a 533×533 µm frame. For the SVZ, cells were counted in a 533×187 µm frame in sections immunostained for BrdU. Cells were manually counted using ImageJ software and summed across the nine images per OB for statistical comparison between animals. The aRMS was defined as the DCX positive area between the OB and the bend between the horizontal and vertical limb of the RMS, whereas the pRMS was defined as the DCX positive are between this bend and the SVZ. Thickness measurements were carried out in a 100 µm distance from the SVZ for the pRMS and a 500 µm distance from the OB for the aRMS. GFAP area measurements in the RMS were done relative to the area covered by DCX staining and results are given as percentage of DCX-positive area. Analysis of the angle of migration in electroporated cells was performed using ZEN software (ZEN Blue Edition, Zeiss, Jena, Germany). A straight line parallel to the RMS main direction, as defined by PSA-NCAM staining, was drawn and the angle between this line and the vector of the cellular leading process was measured for each electroporated (i.e. RFP^+^) cell.

### Statistical analyses

Statistical analysis were made with Prism 5 (GraphPad Inc., La Jolla, USA). Values in all graphs are shown as means±standard error of the mean (s.e.m.). Student's *t*-test and ANOVA were used to test statistical significance, assuming a two-tailed distribution and two-sample unequal variance. A *P*-value below of 0.05 was considered as statistically significant.

## Supplementary Material

Supplementary information
