# Supplementary material for: Cell-autonomous role of GFRα1 in the development of olfactory bulb GABAergic interneurons
Source: Biol Open. 2018 May 1;7(5):bio033753. doi: 10.1242/bio.033753 (PMC5992528; doi:10.1242/bio.033753)
Supplement: Supplementary information [file biolopen-7-033753-s1.pdf]

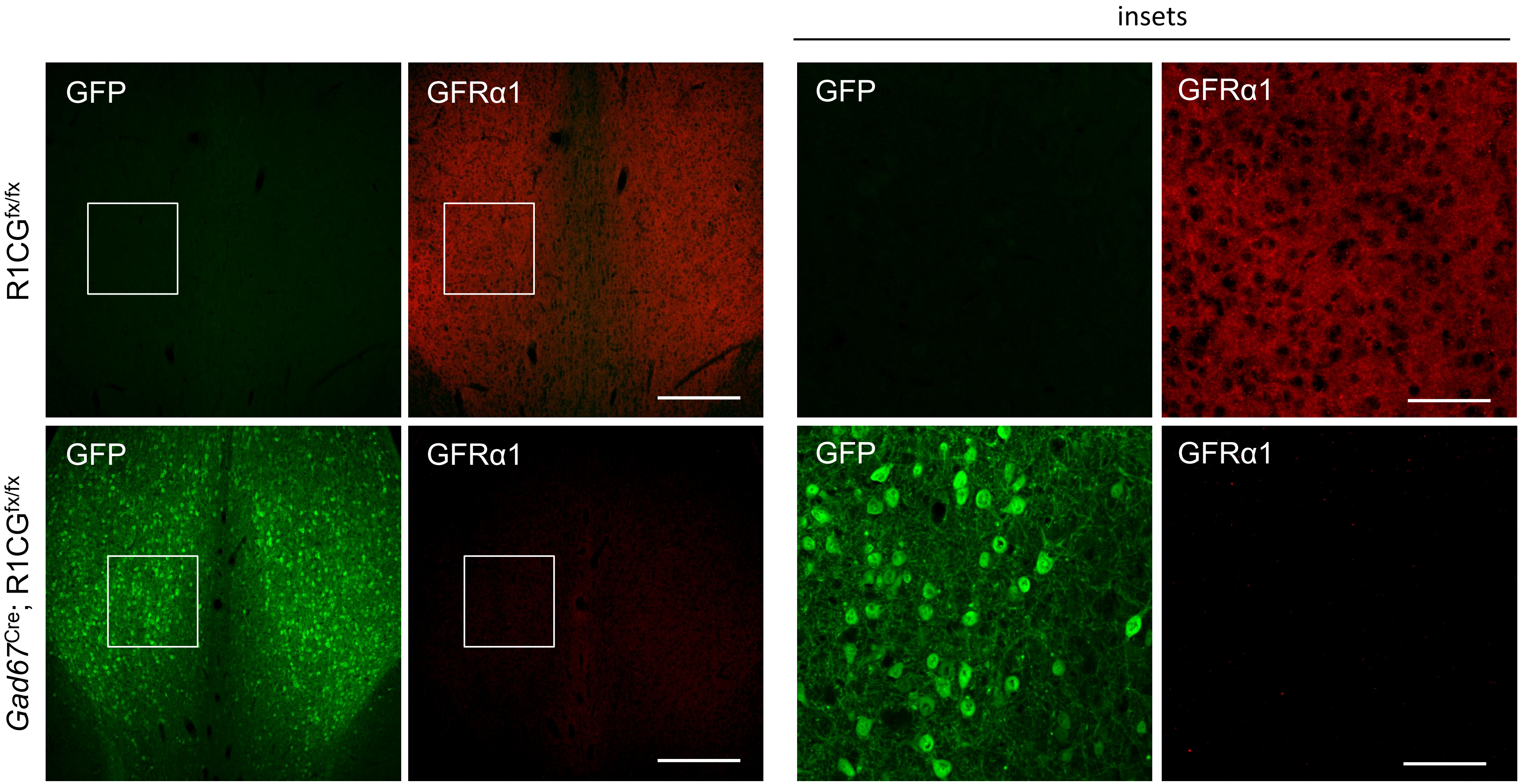

Supplementary Figure 1. Loss of GFRα1 expression in septum of R1CG mice after Cre-mediated recombination driven by *Gad67*^{Cre}

Images derived from the septal area of 7-week old mice. GFP is expressed from the *Gfra1* locus after recombination. Scale bars, 300 μm (leftpanels), 75μm (insets).

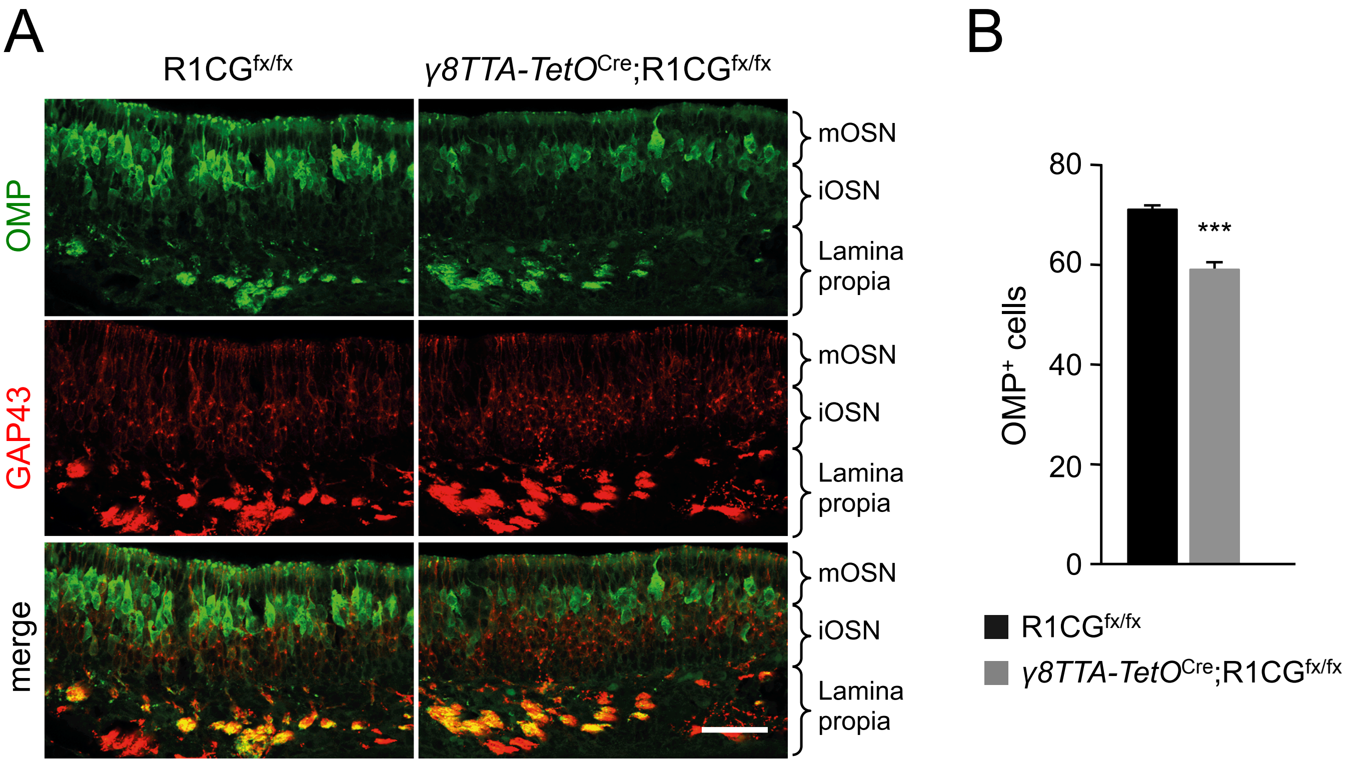

Supplementary Figure 2. Loss of mature OSNs in olfactory epithelium of *γ8TTA-TetO*^{Cre};*R1CG*$^{fx/fx}$ conditional mutant mice

(A) Representative images of olfactory epithelium of P56 *γ8TTA-TetO*^{Cre};*R1CG*$^{fx/fx}$ conditional mutant and *R1CG*$^{fx/fx}$ control mice immunostained for OMP (green, marking mature OSNs) and GAP43 (red, marking immature OSNs). OSN axon bundles can be seen in the lamina propria. Scale bar, 50 μm.

(B) Quantification of mature OSNs (mOSNs) in the olfactory epithelium of P56 *γ8TTA-TetO*^{Cre};*R1CG*$^{fx/fx}$ conditional mutant and *R1CG*$^{fx/fx}$ control mice. N = 6 mice per group. ***, $p < 0.0005$.

Biology Open • Supplementary information

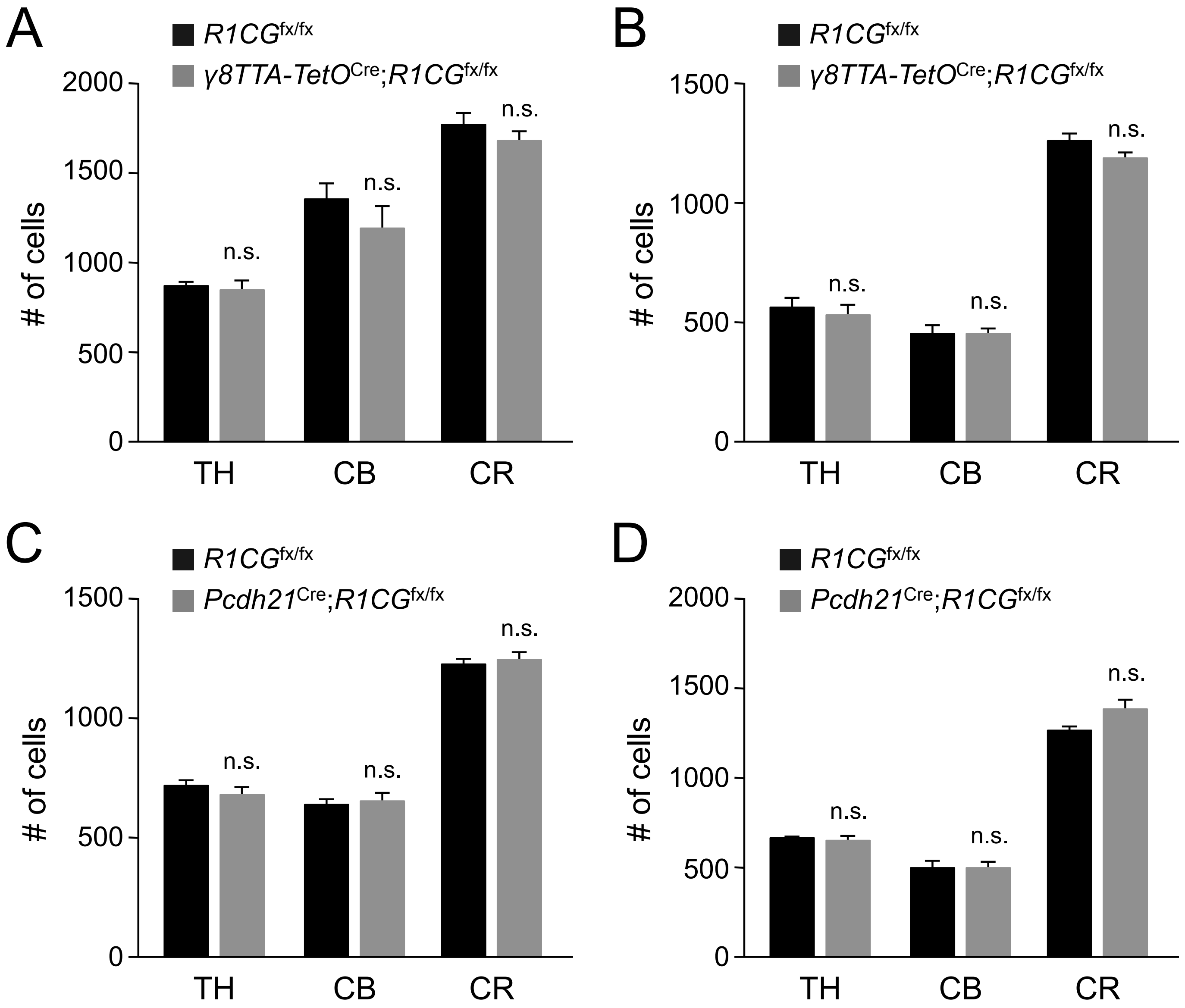

Supplementary Figure 3. No loss of OB GABAergic interneurons in conditional mutants lacking GFRα1 in OSNs or projection neurons

(A, C) Quantification of the number of cells expressing TH, Calbindin (CB) and Calretinin (CR) in the OB of newborn *γ8TTA-TetO*^{Cre};*R1CG*$^{fx/fx}$ (A) and *Pcdh21*^{Cre};*R1CG*$^{fx/fx}$ (C) conditional mutants and R1CGfx/fx control mice. The values represent total number of cells in fields encompassing the entire OB. N = 3 mice per group; n.s., not significantly different.

(B, D) Quantification of the number of cells expressing TH, Calbindin (CB) and Calretinin (CR) in the OB of P56 *γ8TTA-TetO*^{Cre};*R1CG*$^{fx/fx}$ (B) and *Pcdh21*^{Cre};*R1CG*$^{fx/fx}$ (D) conditional mutants and R1CGfx/fx control mice. The values represent number of cells counted in the glomerular layer of the medial surface of the OB (see Materials and Methods for details). N = 3 mice per group; n.s., not significantly different.

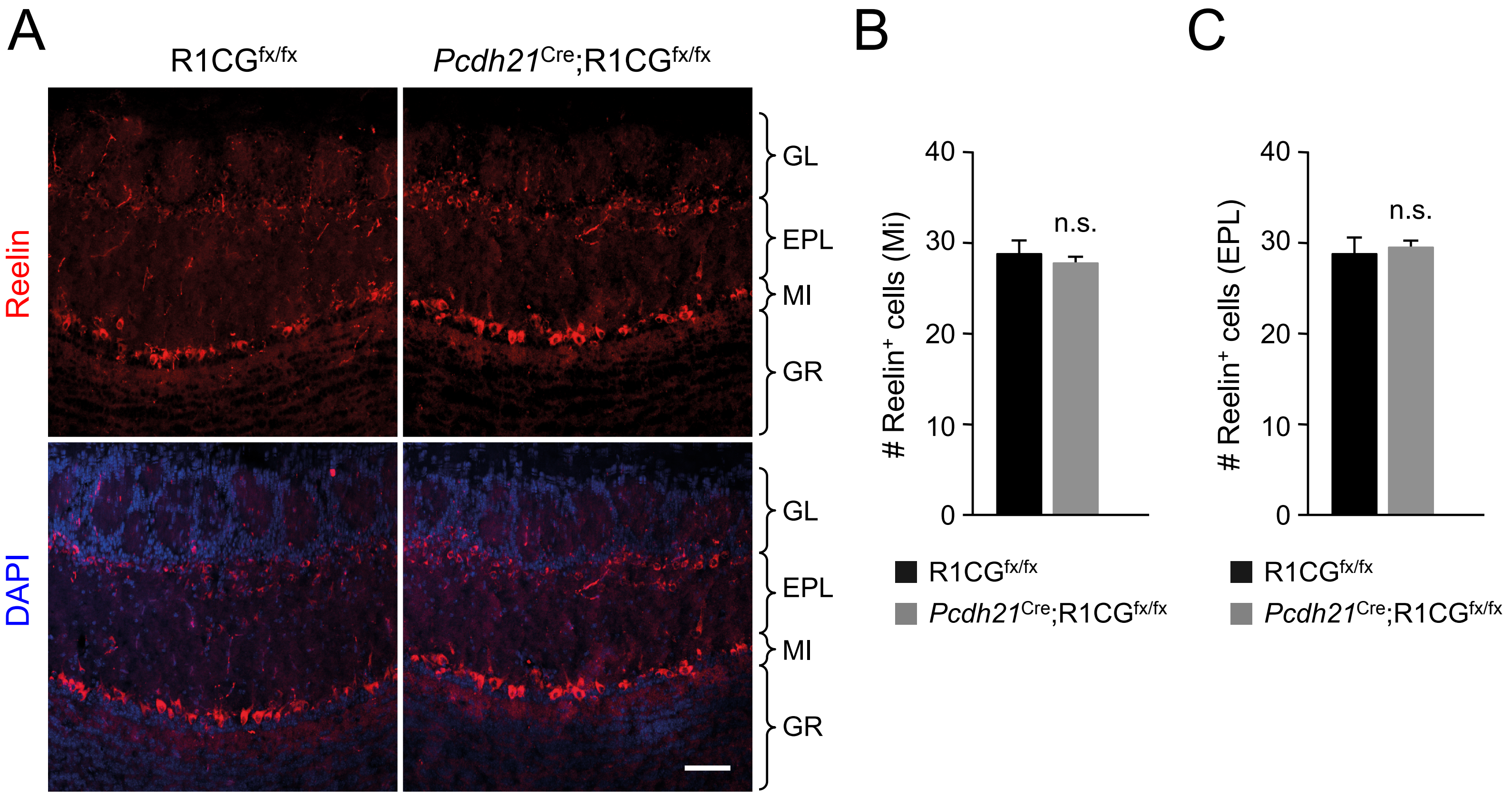

Supplementary Figure 4. No loss of projection neurons in the OB of *Pcdh21*^{Cre};*R1CG*$^{fx/fx}$ conditional mutant mice

(A) Representative images of the OB of P56 *Pcdh21*^{Cre};*R1CG*$^{fx/fx}$ conditional mutant and *R1CG*$^{fx/fx}$ control mice immunostained for Reelin (red), marking mitral cells in Mi layer and tufted cells in the EPL. GL, glomerular layer; EPL, external plexiform layer; MI, mitral cell layer; GR, granule cell layer; Scale bar, 100 μm.

(B, C) Quantification of Reelin^{+} cells in the mitral cell layer (B), i.e. mitral cells, and in the external plexiform layer (C), i.e. external tufted cells, of the OB of P56 *Pcdh21*^{Cre};*R1CG*$^{fx/fx}$ conditional mutant and *R1CG*$^{fx/fx}$ control mice. N = 5 mice per group. n.s., not significantly different.

Biology Open • Supplementary information

Biology Open (2018): doi:10.1242/bio.033753: Supplementary information

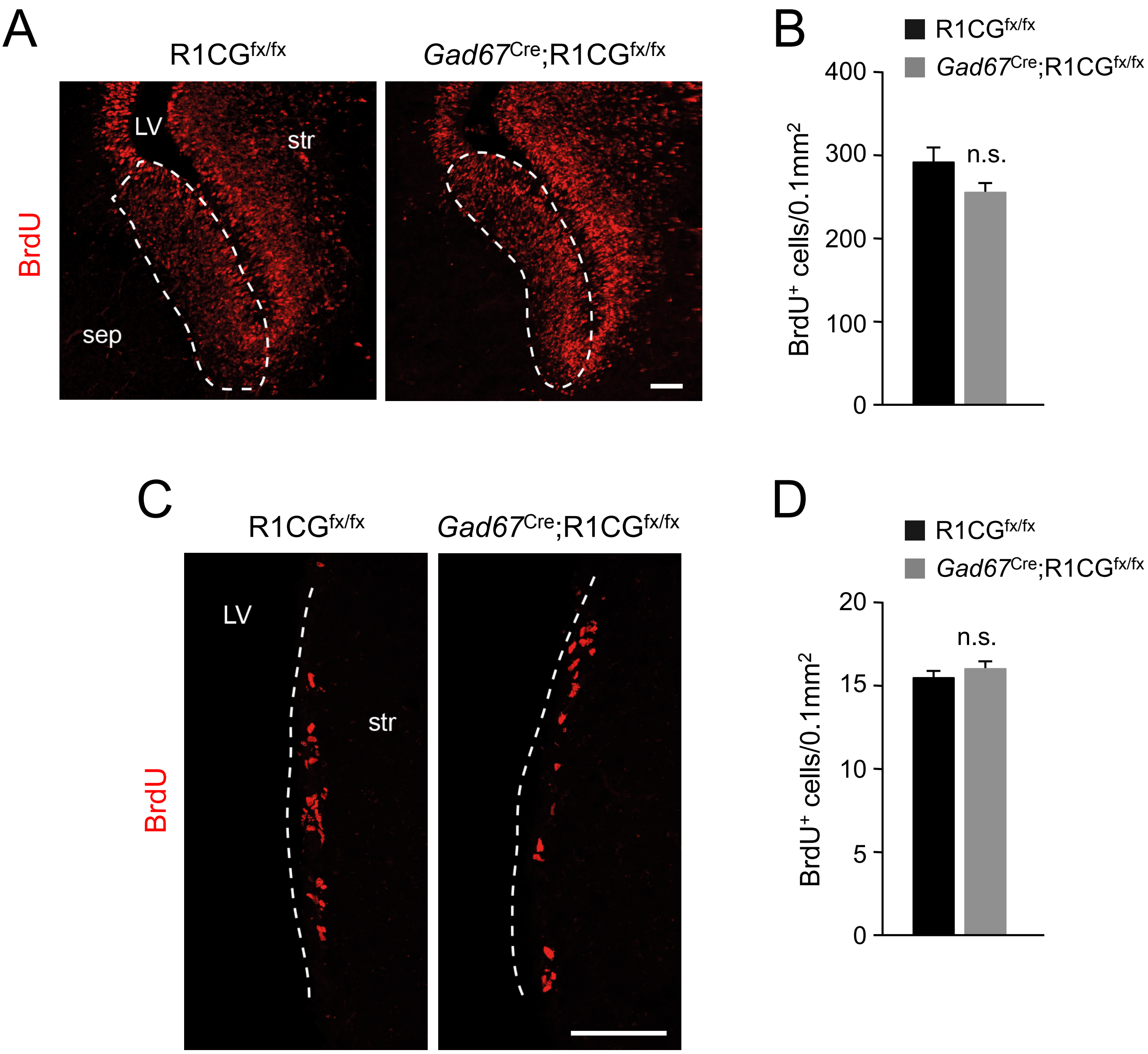

Supplementary Figure 5. Normal proliferation of precursors in embryonic septum and adult SVZ in conditional mutant mice lacking GFRα1 in GABAergic cells

(A) Representative images of BrdU immunostaining from the septal ventricular zone (VZ, dashed line) of E16.5 *Gad67*^{Cre};*R1CG*$^{fx/fx}$ conditional mutant and *R1CG*$^{fx/fx}$ control embryos 30 min after a BrdU injection. LV, lateral ventricle; sep, septum; str, striatum. Scale bar, 100 µm.

(B) Quantification of BrdU positive cells in the septal VZ of septum of E16.5 *Gad67*^{Cre};*R1CG*$^{fx/fx}$ conditional mutant and *R1CG*$^{fx/fx}$ control embryos 30 min after a BrdU injection. N = 5 embryos per group. n.s., not significantly different.

(C) Representative images of BrdU immunostaining from the subventricular zone (SVZ) of the lateral ventricle wall of P56 *Gad67*^{Cre};*R1CG*$^{fx/fx}$ conditional mutant and *R1CG*$^{fx/fx}$ control mice 1 hour after a BrdU injection. LV, lateral ventricle; str, striatum. Scale bar, 100 µm.

(D) Quantification of BrdU positive cells in the SVZ of the lateral ventricle wall of P56 *Gad67*^{Cre};*R1CG*$^{fx/fx}$ conditional mutant and *R1CG*$^{fx/fx}$ control mice 1 hour after a BrdU injection. N = 5 embryos per group. n.s., not significantly different.

Biology Open • Supplementary information

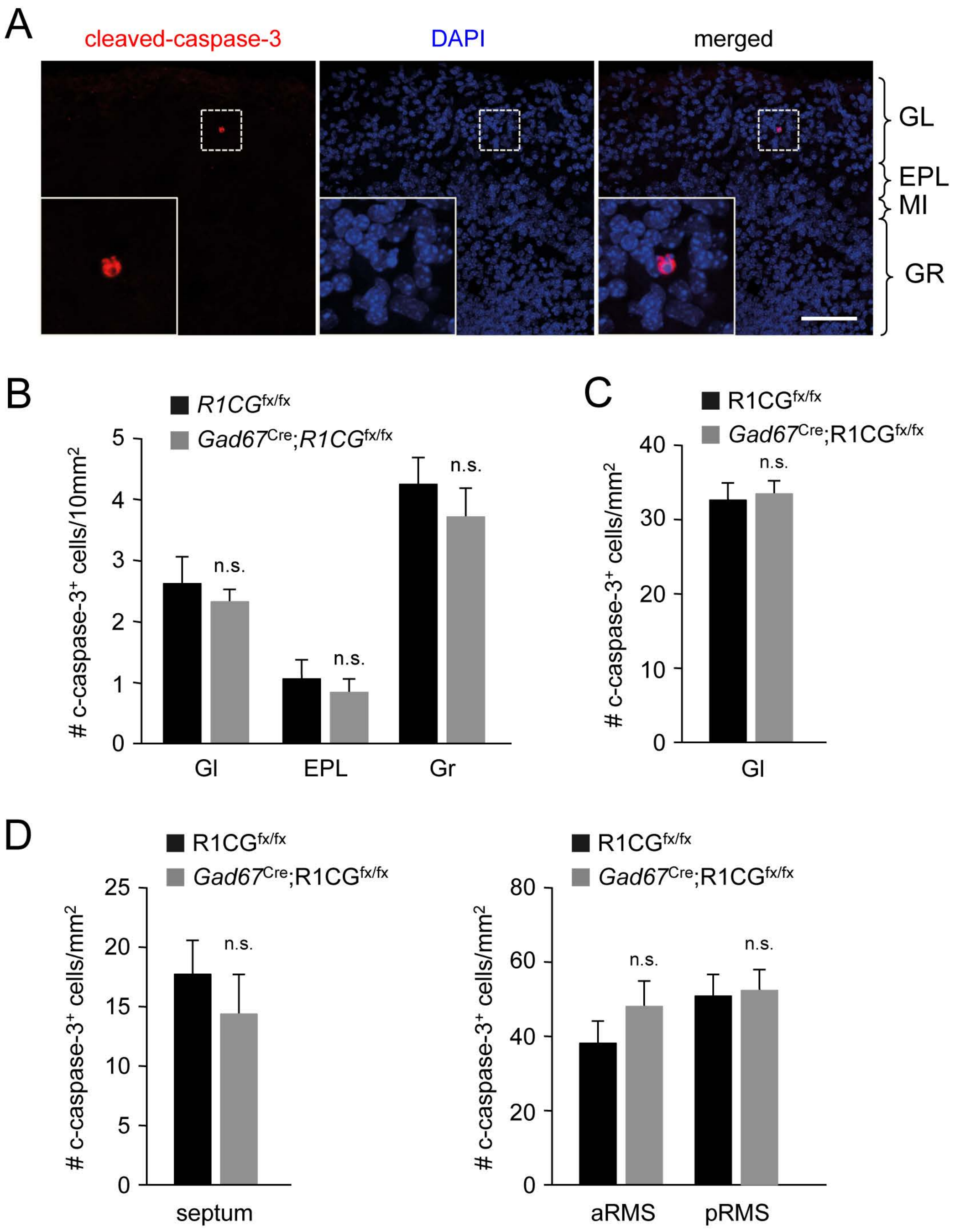

Supplementary Figure 6. Unaltered cell death in conditional mutant mice lacking GFRα1 in GABAergic cells

(A) Representative images of immunostaining for cleaved caspase-3 (red) and DAPI (blue) in the OB of newborn wild type mice. Insets show high magnification of boxed areas. Scale bar, 50 μm.

(B) Quantification of cleaved-caspase-3 in the OB of E16.5 *Gad67*^{Cre};*R1CG*$^{fx/fx}$ conditional mutant and *R1CG*$^{fx/fx}$ control embryos. GL, glomerular layer; EPL, external plexiform layer; GR, granule cell layer; M, mitral cell layer. N = 5 embryos per group. n.s., not significantly different.

(C) Quantification of cleaved-caspase-3 in the medial surface of the glomerular layer of P56 *Gad67*^{Cre};*R1CG*$^{fx/fx}$ conditional mutant and *R1CG*$^{fx/fx}$ control mice. Gl, glomerular layer. N = 5 mice per group. n.s., not significantly different.

(D) Quantification of cleaved-caspase-3 in the septum of E16.5 *Gad67*^{Cre};*R1CG*$^{fx/fx}$ conditional mutant and *R1CG*$^{fx/fx}$ control embryos. N = 5 embryos per group. n.s., not significantly different.

(E) Quantification of cleaved-caspase-3 in the anterior and posterior RMS of P56 *Gad67*^{Cre};*R1CG*$^{fx/fx}$ conditional mutant and *R1CG*$^{fx/fx}$ control mice. N = 5 mice per group. n.s., not significantly different.

Biology Open • Supplementary information

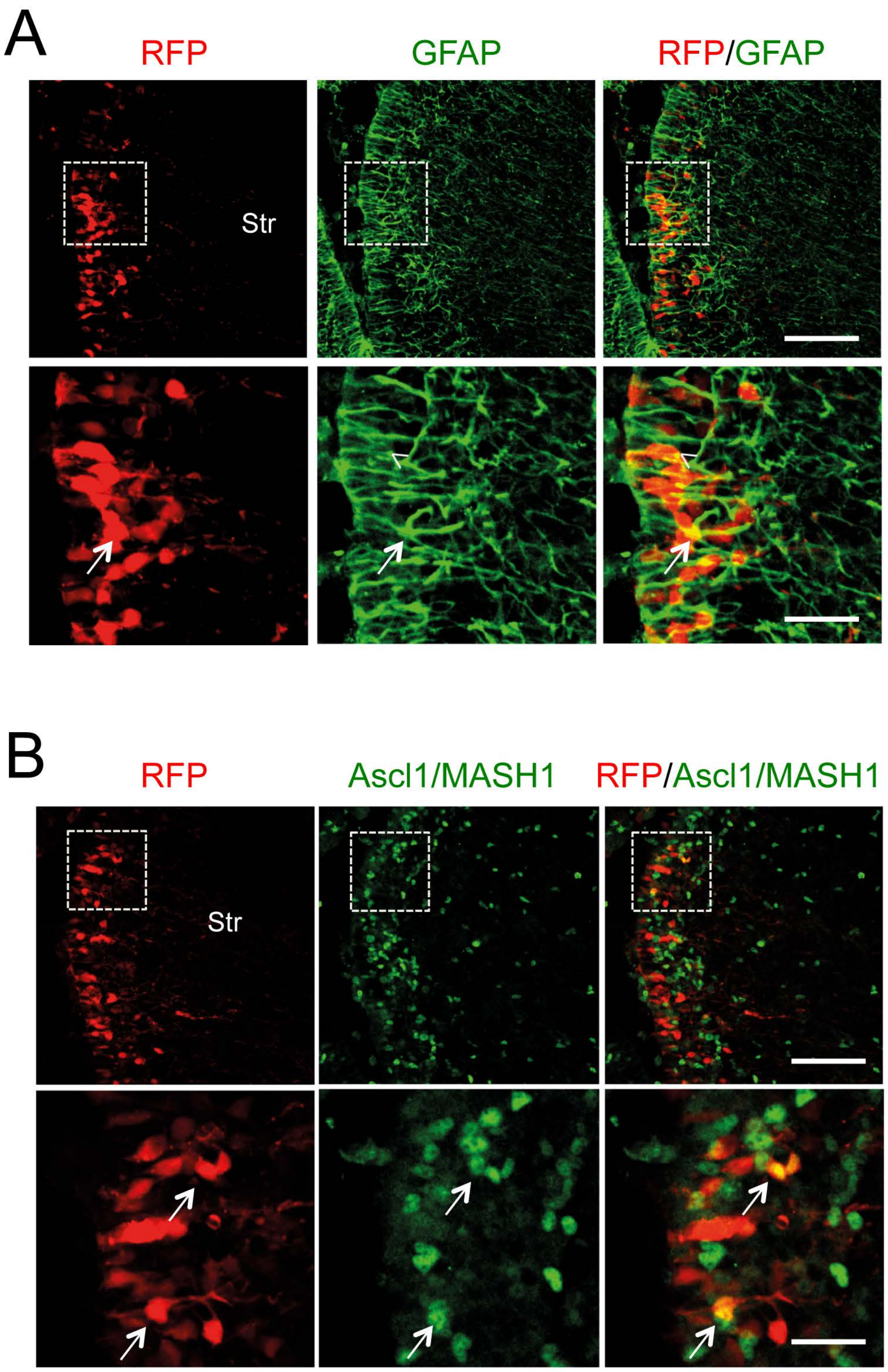

Supplementary Figure 7. Targeting of precursor cells in the ventricular wall of neonate mice by *in vivo* electroporation

(A, B) Representative images of expression of RFP (red, marking electroporated cells) and GFAP (A) or Ascl1/MASH1 (B) (green, marking type C transit amplifying cells) in the SVZ two days after ventricular electroporation of a RFP-expressing plasmid in new born wild type mice. Bottom row panels show high magnification of boxed areas. Arrows indicate double positive cells. Scale bar, 50 µm (upper panels), 25 µm (lower panels).

Biology Open • Supplementary information
